# Exploring chronic disease in Bolivia: A cross-sectional study in La Paz

**DOI:** 10.1371/journal.pone.0189218

**Published:** 2018-02-07

**Authors:** Patricia Abbott, Tanima Banerjee, Amparo Clara Aruquipa Yujra, Boqin Xie, John Piette

**Affiliations:** 1 University of Michigan School of Nursing; Ann Arbor, Michigan, United States of America; 2 Institute for Healthcare Policy and Innovation, University of Michigan; Ann Arbor, Michigan, United States of America; 3 Universidad Catolica Bolivıana; La Paz, Bolivia; 4 Fudan University School of Nursing, Shanghai, China; 5 School of Public Health, University of Michigan, Ann Arbor, Michigan, United States of America; Istituto Di Ricerche Farmacologiche Mario Negri, ITALY

## Abstract

**Purpose:**

This study seeks to develop an understanding that can guide development of programs to improve health and care for individuals with Non-Communicable Diseases (NCDs) in La Paz, Bolivia, where NCDs are prevalent and primary care systems are weak. This exploratory investigation examines the characteristics of chronic disease patients in the region, key health related behaviors, and their perceptions of the care that they receive. The longer-term goal is to lay groundwork for interventional studies based on the principles of the Chronic Care Model (CCM).

**Subjects and methods:**

The study is based on two surveys of adults (> 18 years old) administered in 2014 in La Paz, Bolivia. A total of 1165 adult patients participated in the first screening survey. A post-screening second survey, administered only on those who qualified based on Survey 1, collected more detailed information about the subjects’ general health and health related personal circumstances, several health behaviors, health literacy, and their perceptions of care received. A final data set of 651 merged records were used for analysis.

**Results:**

Characteristic of a low-income country, the majority of participants had low levels of education, income, health literacy and high rates of under/unemployment. Nearly 50% of participants reported 2 or more NCDs. Seventy-four percent (74%) of respondents reported low levels of medication adherence and 26% of the population was found to have an undiagnosed depressive disorder. Overall, the perception of care quality was low (60%), particularly in those under the age of 45. Significant relationships emerged between several sociodemographic characteristics, health behaviors, and perceptions that have major implications for improving NCD care in this population.

**Conclusions:**

These findings illustrate some of the challenges facing low-income countries where reversing the tide of NCDs is of great importance. The prevalence of NCDs coupled with challenging social determinants of health, poor medication adherence, low health literacy, and perceptions of low quality of healthcare highlight several areas of opportunity for intervention.

## Introduction

Non-communicable diseases (NCDs)—particularly hypertension, diabetes, depression, and lung disease—are significant and pressing global health issues. The prevalence of NCDs has doubled in low and middle income countries (LMIC) in recent decades [1, 2], and LMICs represent 84% of the burden of years living with NCD-related disability worldwide [3]. NCDs are now the principal cause of death in the developing world, accounting for twice as many deaths as tuberculosis, malaria, and HIV combined [4]. Further complicating the health landscape, many LMICs exhibit the double load of non-communicable and infectious disease, placing devastatingly heavy burdens on their emerging economies [5].

Latin American and Caribbean (LAC) nations are particularly challenged by the increasing incidence and prevalence of NCDs. By 2035, the number of people with diabetes in Central and South America is predicted to grow from 24.1 million to 38.5 million, an increase of 60%, in contrast to a projected increase of 37% in North America and the Caribbean [6]. A recently released report from Pan American Health Organization (PAHO) reveals that 80% of all deaths in the PAHO region are now attributed to NCDs [7]. This same report asserts that over 59% of all persons in the PAHO region are considered to be obese, with physical inactivity 1.5 times greater than anywhere else in the world. These statistics are compounded by an expected doubling of the overall population in the region by 2040, with the fastest growing segment of the population those aged 60 and older [7]. By 2030, Latin America is projected to have the highest burden and prevalence of overweight and obese people in the world [8].

The linkage of NCDs to well-established social determinants of health is complex. Income levels, age, behaviors, policies and governance, gender, ethnicity, and a myriad of other social risk factors vary greatly across the South American continent. The interaction between the social risk factors with intermediary determinants, such as health system structure and behavioral/psycho-social issues, further complicate the landscape. Information systems in many developing nations of LAC are poor [9, 10]. As a result, the data needed to understand the population, its characteristics, and its use of health services in relation to NCDs—is lacking. While it is known that the impact of NCDs can be reduced through lifestyle changes such as improved diet overall and reduction of sodium more specifically, mental health treatment, smoking cessation, and increased physical activity[11], implementation of such changes in LACs requires enhanced public and community health efforts coupled with concerted efforts to improve primary care. The enormous burden that uncontrolled NCD growth is already exerting on LAC national budgets and its impact on sustainable economic growth[12, 13] requires that these issues be better understood in order to most effectively intervene.

A first order of business on many national agendas in LAC has become stemming the NCD tide. This is particularly noted in Bolivia which holds the “35 year-old sad record of the worst social and health indicators of Latin America.”[14], and like many nations in the world, is home to a rapidly aging population. In the Americas, only Haiti ranks lower than Bolivia in maternal and under-five year old mortality rates,[2] and Bolivia was ranked 27^th^ out of 33 of all Latin America and Caribbean nations on the Human Development Index[15].

Issues faced by the Bolivian health care system in regards to healthcare coverage, financing, and protection for the most vulnerable members of society are similar to those found in other LMICs around the globe. The percentage of funding allocated by the Bolivian government for health services, social security, and public insurance is low, comprising only 9.5% of the yearly total governmental expenditures, far below the global average of 15%.[16] Bolivia has, however, made important progress towards improving healthcare over the last decade, expending 5.8% of its GDP on healthcare which (while below the industrialized world rates of 9.2%) exceeds that of comparable low income countries rates of 4.5%.[16]. Additionally, in 2008 the Bolivian government established the Unified Family, Community and Intercultural Health System (SAFCI) with the goal of creating an integrated, intersectoral and intercultural primary health care system in the country. Incorporated into the SAFCI is a Universal Maternal and Child Health Insurance plan (SUMI) and Health Insurance for Older (SSPAM), which are specifically designed to address disparities in maternal/child and elderly care, respectively.

These are important and commendable movements towards improving health and healthcare for the Bolivian people, however there is much work left to be done. At present, only a small fraction of the population has access to any type of comprehensive health and social services; safety net programs such as disability, pensions, and catastrophic coverage are exceedingly rare; and the national Social Security scheme reaches only 1/3 of the population.[16] Because over 80% of all Bolivians are part of the informal economy (meaning no benefits, insurance, contributions to Social Security or pensions) a vast majority of Bolivians are left to fend for themselves, paying out of pocket or not receiving care at all. [17] Finally, while the governmental efforts have reduced rate of maternal and child mortality, a recent systematic review and analysis of primary health services in Bolivia found so few rigorous studies that ascertaining any changes in health status due to the these national health initiatives to improve services was impossible [18]. The fragmented state of information systems and a paucity of specific health data are major impediments to evaluation and monitoring efforts in Bolivia, a fact pointed out by several authors.[10, 18, 19]

The Pan American Health Organization (PAHO), in addressing the mismatch between the rapidly increasing burden of NCDs and the way in which many LAC healthcare systems are equipped to deal with them, has developed a “Regional Strategy for Chronic Disease Prevention and Control”. [20]. The PAHO regional strategy specifically addresses the management and integration of NCDs into primary care, includes directives for robust information technology for data collection and analysis, and calls for adaptations of health systems for differences in culture, health behaviors, languages, and delivery models. The Chronic Care Model (CCM), specifically adopted by PAHO as the framework for this effort, is designed to guide the provision of patient-centered, coordinated, planful and proactive care for patients with chronic disease and is used widely around the globe. [21]

### Study goals and questions

In light of the circumstances noted prior, this study seeks to develop an understanding that can guide development of supportive programs to improve health and care for individuals with NCDs in La Paz, Bolivia: a site of established collaborative efforts between the University of Michigan and La Paz el Servicio Departamental de Salud (SEDES). Like other areas of the Andean region, La Paz is marked by high prevalence of NCDs, poverty, scarce data, an aging population, and weak primary care systems. This exploratory investigation examines the characteristics of chronic disease patients, key health related behaviors, and their perceptions of the care that they receive in the area. The longer-term goal is to lay groundwork for patient-centered, evidence-based, and contextually appropriate NCD interventions in support of the PAHO CCM-based regional strategy.

This exploratory study explores relationships among four categories of variables in a sample of chronic disease patients in La Paz: (1) sociodemographics (demographics, education, income, and health literacy); (2) disease status (chronic disease types and number); (3) health related behaviors (alcohol use and medication compliance); and (4) patient’s perceptions of health care quality (fundamental to the CCM). We specifically address the following questions considered most important to this initial portrayal of the survey results:

1. What are the characteristics of this sample across the four categories of variables (socio-demographics, disease status, health related behaviors, and perceptions of care quality)?
2. To what extent do depression, the health related behaviors of alcohol use and medication adherence, number of NCDs, and patients’ perception of care quality relate to sociodemographic characteristics of the sample?
3. To what extent do the health related behaviors of alcohol use, medication adherence, and perceptions of care relate to the five most prevalent chronic diseases and their frequency of co-occurrence?

## Methods

### Study design

The study is based on two surveys of adults (> 18 years old) administered in June and August of 2014 in La Paz, Bolivia. The first survey obtained a cross-sectional portrayal of the population including various sociodemographics and presence of chronic health conditions; the results were used primarily to select subjects who would receive the second survey. The second survey, administered only to patients who met the inclusion criteria discussed below, employed previously validated scales[22] to explore health literacy, the health related behaviors of medication adherence and alcohol use, and CCM perceptions of care quality. The scales we employed were used in several prior studies in La Paz, by a highly experienced team of Bolivian and US researchers. All tools have been validated in Spanish speaking populations. Psychometric properties for the Spanish language versions are reported below. Prior papers from this team in La Paz have focused on respondents’ use of mobile phones and the potential of mHealth interventions for improving chronic disease self-management support.[22–25] The results of this study will allow us to further pinpoint distinct dimensions that could be wound into future mHealth and CCM-based interventions for specific NCDs in La Paz.

Data from both surveys were analyzed to address the three research questions guiding this report. Written informed consent was obtained from all participants. The study was approved by the University of Michigan Institutional Review Board and the la Universidad Cathólica Boliviana UCB ethics committee.

### Survey procedures and study sample

Prospective subjects were approached during routine primary care clinic visits to two public hospitals and one private hospital in La Paz, one public hospital in the sister city of El Alto, and at two public health fairs in La Paz. Assessments of all participants sociodemographic characteristics revealed no significant differences in subject characteristics from any of the sites regardless of type (public, private, clinic, or location). A team of University of Michigan students who were fluent in Spanish, as well as surveyors who were Bolivian clinicians and medical students affiliated with the la Universidad Cathólica Boliviana (UCB), verbally administered both surveys. Native Spanish speakers translated, as needed, and tested all surveys materials prior to patient recruitment as reported elsewhere.[22]

**Fig 1:**
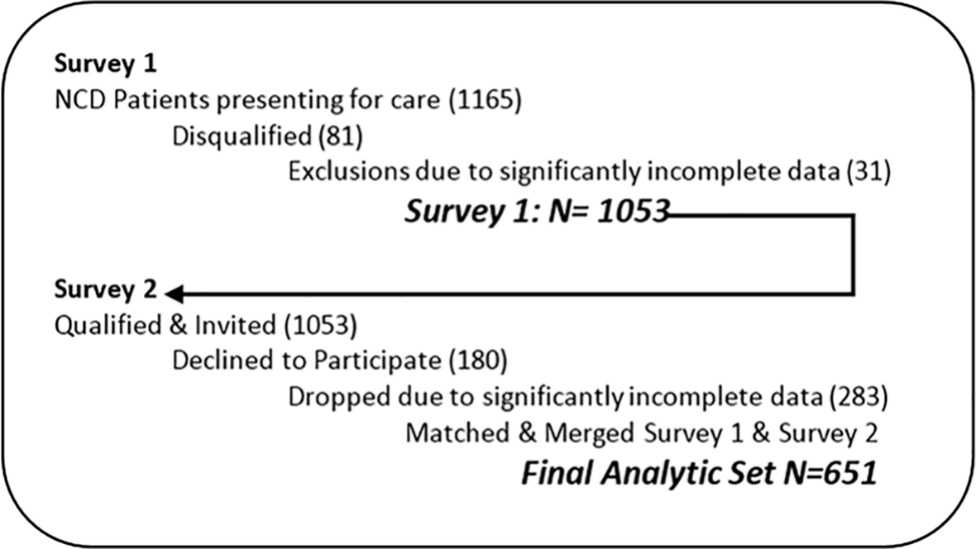
Study design reflecting recruitment, inclusion, and exclusion.

A total of 1165 adult patients participated in the first screening survey. Patients with an immediately life-threatening health problem such as cancer, those who were visiting the clinic for an urgent health problem, or those with severe mental illness as reported by their clinical team were excluded from any further study engagement (n = 81). See Fig 1. Upon subsequent inspection, 31 first-round surveys were found to be significantly incomplete, leaving an analytic sample of 1053. Subjects from survey 1 who met inclusion criteria (one or more chronic conditions, or had systolic blood pressure exceeding 140 mm/hg, or who were classified as “depressed” with a score of 3–6 via the PHQ2 depression scale)[26] were invited to participate in the second survey.

The second survey collected more detailed information about the subjects’ general health and health related personal circumstances, several health behaviors, health literacy, and their perceptions of the quality of the care they received. Six percent (6%) of those who qualified and invited declined to participate (180 of 1053). Records from Survey 1 and Survey 2 were then merged, creating a final data set for the study. Subjects who completed less than 75% of the items in Survey 2 (n = 283) were dropped entirely from the dataset, leaving 651 subjects in the final analytical sample for Survey 2. Any individual survey scale that contained less than 75% of completed items was excluded from the analysis of that particular item.

### Measurements

The variables in the study were assessed using two separate surveys. Survey 1 included a socio-demographic questionnaire, general questions about NCDs, and medications currently taken. Survey 2 gathered more detail about the patient’s health condition and included several standardized scales that are described below. All surveys and assessment tools used in this study, with the exception of the Morisky Medication Adherence Scale, are in the public domain. For permissions or questions related to the Morisky widget, scales, translations, and training, contact MMAS Research at 14725 NE 20th Street; Bellevue, WA 98007.

#### Health literacy

Health literacy was assessed with a single question based on the work of Wallace, Rogers, Roskos, Holliday, & Weiss (2006) [27]. Subjects were asked (in Spanish): “How confident are you filling out medical forms by yourself?” Respondents who answered “somewhat”, “a little bit” or “not at all” to this question were categorized as having low health literacy. The Wallace single question approach, when used in clinic populations, has been shown to be accurate in detecting limited/marginal health literacy skills (AUROC of 0.79; 95% CI = 0.74 to 0.83) and limited (AUROC of 0.82; 95% confidence interval [CI] = 0.77 to 0.86) health literacy skills [27].

#### Depression

The eight item version of the Patient Health Questionnaire (PHQ8) measures severity of depression, with reliability measures (Cronbach’s alpha) at .77 for the Spanish version.[28] Patients are asked about specific feelings or mood occurrences over the past week with anchors from “0 = not at all” to “3 = nearly every day”. Patients are considered to be depressed if they answered at least four questions as “2 = more than half the days” or “3 = nearly every day”[28].

#### Medication adherence

The Morisky 8-item medication adherence scale is used to detect patients with a medication adherence problem and to monitor adherence over the course of treatment. [29] The Morisky scale has been translated into numerous languages including Spanish, used extensively, and validated for adherence to medications for hypertension, diabetes, long-term infectious diseases, chronic renal disease, and osteoporosis [29].^.^ In this study, the Morisky was administered to those with the reported diagnosis of hypertension or diabetes (N = 438). Respondents with a score of greater than 2 on the Morisky are considered to have a low level of medication adherence, while those with a score of 1 or 2 are considered to be moderately adherent, and a score of zero denotes a high level of adherence (Cronbach alpha = .83). [29]^.^ Because of small numbers of respondents who fell into the high adherence categories in this study, these respondents were aggregated into a moderate/high category for analytic purposes.

#### Alcohol use

The AUDIT tool was developed by the World Health Organization (WHO) as a reliable (alpha = .86) and internationally relevant way to screen individuals for excessive alcohol consumption and to assist in a brief assessment of alcohol ingestion behaviors.[30] Each item on this scale is scored between a 0 and a 4, and the items are summed to obtain a total score. Per the scoring guidelines, those with total scores of between 1–7 represent a “low” level of alcohol use; a total score of 8–15 represents a “medium” level of an alcohol use, and a score of 16 or higher represents a “high” level of alcohol use. In subjects who denied any alcohol use, a score of zero was recorded. In this analysis, we categorized and reported alcohol use as either none/low (between 0–7) or moderate/high (scores of 8 or greater) per scoring guidelines.

#### Perceptions of care quality

The PACIC measures patients’ perspectives regarding the quality of care they have received [21]. The PACIC includes 20-items organized into five subscales; the 5 subscale scores and the PACIC total score based on all 20 items were employed in this analysis. Each item uses a 1–5 rating scale with higher scores indicating higher perceptions of care quality. We used the Spanish language version of the PACIC, which demonstrates high internal consistency (Cronbach α = 0.87) and test-retest reliability (r = 0.77) [31]. For the purposes of this analysis and as suggested by Boyd et al., (2010), [32] the total PACIC score, computed as averages over all scale items, were categorized into three levels: high-quality of care (score > 4), medium quality of care (score ≥3 and ≤4), or low-quality care (score <3).

### Data analysis

#### Study question 1

What are the characteristics of this unique sample across the four categories of variables (socio-demographics, disease status, health related behaviors, and perceptions of care quality)? To address this question, we report frequencies and percentages for all categories of all relevant study variables reflected in Table 1.

**Table 1:** Sample characteristics.

	n (%)^a^		n (%)^a^
**Gender** (n = 651)		**Health Literacy** (n = 643)	
Male	241 (37)	Low	372 (58)
Female	410 (63)	Normal	271 (42)
**Age** (n = 651)		**Diagnosed Chronic Illnesses** (N = 651)	
<45	206 (32)	Hypertension	228 (36)
45<65	303 (47)	Diabetes	191 (30)
> 65	142 (22)	High cholesterol	148 (24)
		Depression	143 (23)
**Education** (n = 633)	269 (41)	Arthritis	101 (16)
<9 years	177 (27)		
9–11 years	187 (29)	**Number of Chronic Disease** (N = 651)	
12 or more		1 Chronic Disease	332 (51)
		2 Chronic Diseases	150 (23)
**Marital Status** (n = 636)	126 (19)	3 or more Chronic Diseases	169 (26)
Single	380 (58)		
Married/consentual union	130 (20)	**Perceptions of Care Quality** (PACIC; n = 529)	
Divorced/seperated/widowed		Low	316 (60)
		Medium	140 (26)
**Occupation** (n = 650)		High	74 (14)
Blue collar	203 (31)		
White collar	48 (7)	**Alcohol Use** (AUDIT; n = 625)	
Professional	67 (10)	none/low	552 (88)
Other	54 (8)	mod/high	73(12)
Not working/retired	278 (43)		
		**Medication Adherence** (Morisky; n = 438)	
**Weekly Family Income** (n = 455)		low adherence	324 (74)
<600 Bs	297 (65)	mod/high Adherence	114 (26)
600–1200 Bs	115 (25)		
> 1200Bs	48 (10)	**Depression Scale** (PHQ-8; n = 617)	
		depressed	170 (26)
**Distance From Clinic** (n = 598)		not depressed	481 (74)
<1 Km	335 (56)		
1–5 Km	111 (19)		
6 Km or more	152 (25)		

Bs = Bolivian Boliviano. 1 US dollar = 6.91 Bs

^a^ percentage of sample per response

#### Study question 2

To what extent do depression, the number of NCDs, the health related behaviors of alcohol use and medication adherence, and patients’ perception of care quality relate to sociodemographic characteristics of the sample? This analysis uses depression, alcohol use, medication adherence, and the PACIC overall care quality perception as dependent variables. Eight variables reflecting participants’ sociodemographic characteristics are used as the independent measures. For purposes of exploratory analysis, we employed contingency table methods for each bivariate relationship and employed chi-square tests of statistical significance. To gain a better understanding of disease burden by age, a separate bivariate analysis using frequency distribution was undertaken to examine the distribution of the number of NCDs by age. Finally, we conducted logistic regressions using dichotomized outcome variables and sociodemographic characteristics to model independent contributions of multiple variables. Stepwise & backward selection criteria were used to choose the reduced models for each outcome of interest. Variables that are not associated with outcomes (non-significant variables) were excluded from the final models to improve the precision. The final parsimonious logistic regression models were fitted for the four of the health-related behavior outcomes separately to estimate the AORs of the retained independent variables.

#### Study question 3

To what extent do the health related behaviors of alcohol use, medication adherence, and perceptions of care relate to the five most prevalent chronic diseases and their frequency of co-occurrence? In this analysis, we treated alcohol use, medication adherence and overall quality of care perception as dependent variables and chronic disease type and number of NCDs as the independent variables of interest. Logistic regression models were fitted for each of the independent variables to estimate odds ratios for the outcomes separately.

## Results

### Study question 1: Characteristics of the sample

#### Personal characteristics

The sample was 63% female with mean age of 53 ± 15 years. See Table 1. The majority of respondents had an educational level of less than 9 years (41%), with 27% having some high school, and 29% having at least some higher education beyond high school. The majority of the sample were either married or in a consensual union (58%). Forty-three percent (43%) of the 455 who responded were either unemployed or retired, and 31% were employed in blue-collar occupations with 65% reporting combined family incomes of less than $87.00 USD per week. Thirty percent of the sample (n = 196) were either unable or unwilling to answer the income question. Finally, consistent with the urban character of the population, 56% reported living less than 5 kilometers from the clinic where they receive care. The majority of participants (58%) fell into the low health literacy category.

#### Chronic disease states

While all participants had to have a least one chronic disease to be included in the sample, 319 subjects (49%) reported having two or more chronic diseases. Table 1 also reports the five most prevalent diseases, with 36% reporting hypertension and 30% diabetes across the entire sample.

#### Medication adherence

Table 1 illustrates that seventy-four percent (74%) of the 438 respondents reported low medication adherence and 26% reported medium/high levels of medication adherence. The primary contributors to low adherence as measured by the Morisky scale across all participants involved forgetting to take medications (50% of respondents reported forgetfulness) and forgetting to bring medications with them when travelling outside of the home (36%). Thirty percent of respondents reported that they had days in the last two weeks where they had not taken their medicine, and 28% reported that they electively stopped taking medications when they felt that their symptoms were under control.

#### Alcohol use

The AUDIT scale, part of the second survey, was only administered to those who reported any use of alcohol (n = 513). As described earlier, the 112 subjects who reported no use of alcohol did not take the AUDIT and were scored with a zero. After combining the 513 AUDIT results with the 112 who reported no alcohol use (n = 625), 88% were categorized to have low to no level of alcohol use, and 12% were labeled as having medium or high use of alcohol. See Table 1.

#### Perceived quality of care

Of the 651 final subjects, 122 failed to complete the required 75% of the 20 PACIC scale items, resulting in a final PACIC sample size of 529 as described earlier in the Survey Procedures section. The mean of the total PACIC score in this sample was 2.73 (SD = 0.99), reflective of an overall perception of low quality of care (any score less than 3). The highest mean score found within the 5 subscales was for the decision support/ delivery system design subscale (3.65 ±1.20), characterized by questions such as “Shown how what I did to take care of myself influenced my condition” and “given a written list of things I should do to improve my health.” Subscales with low mean scores included problem-solving/contextual counseling (3.06±1.37), e.g., “asked how my chronic condition affects my life”; goal setting (2.77±1.18), e.g., “given a copy of my treatment plan”; and patient activation (2.38±1.30), “asked for my ideas when we made a treatment plan”. The lowest score reported by patients was in follow-up/ coordination (Mean = 2.08, SD = 1.10), e.g., “contacted after a visit to see how things were going”. More than 50% of respondents rated follow-up, goal setting, and patient activation with a score of less than the cut-point of 3, reflecting a perception of low quality. For purpose of the overall analysis, the total score was transformed into the three categories of low, medium, and high quality perceptions as described earlier. Based on the transformation, sixty percent (60%) of the subjects (n = 316) rated the quality of their care as low as shown in Table 1.

### Study question 2: Sociodemographic correlates of health

Research question 2 results are reflected in Tables 2, 3 and 4. In Table 2 we report the frequency distributions & p-values from the chi-square tests for the sociodemographic characteristics stratified by each outcome category. Nine of the 32 bivariate relationships, are significant at p < .05. Table 3 displays the proportions of three age groups by number of NCDs. Table 4 show the results of multiple logistic regressions used to estimate adjusted odds ratios (AORs) for which all significant relationships found earlier in our chi-square (Table 2) remained except that of marital relationship and alcohol use.

**Table 2:** Sociodemographic characteristics by health behaviors (chi-square tests).

	PHQ n *(%)*^*a*^			PACIC n (%)^*a*^				AUDIT n (%)^a^			Morisky n (%)^a^		
	*Depressed*	*Non-Depressed*		*low*	*medium*	*high*		*None/Low*	*Moderate/High*		*Low*	*Medium/High*	
Socio-demographics (n = 651)			*P-Value*				*P-Value*			*P-Value*			*P-Value*
**Age**			*0*.*675 (n = 617)*				***0*.*01 (n = 529)***			**.*005 (n = 625)***			**.*001 (n = 438)***
<45	55 (27)	151 (73)		103 (73)	26 (18)	12 (9)		165 (83)	35 (17)		90 (82)	20 (18)	
45<65	82 (27)	221 (73)		137 (54)	72 (28)	45 (18)		260 (90)	29 (10)		159 (77)	48 (23)	
> 65	33 (23)	109 (77)		76 (57)	42 (31)	16 (12)		127 (93)	9 (7)		75 (62)	46 (38)	
**Gender** (n = 651)			***0*.*01 (n = 651)***				.*848 (n = 529)*			**.*0001 (n = 625)***			.*917 (n = 438)*
Male	48 (20)	193 (80)		124 (61)	54 (27)	26 (13)		185 (79)	48 (21)		124 (74)	43 (26)	
Female	122 (30)	288 (70)		192 (59)	86 (27)	47 (15)		367 (94)	25 (6)		200 (74)	71 (26)	
**Health Literacy** (n = 643)			.*156 (n = 643)*				.*355 (n = 521)*			*0*.*246 (n = 617)*			**.*05 (n = 431)***
Low	105 (28)	267 (72)		183 (62)	75 (25)	37 (13)		309 (87)	46 (13)		186 (78)	52 (22)	
Normal	63 (16)	208 (77)		127 (56)	63 (28)	36 (16)		236 (90)	26 (10)		133 (69)	60 (31)	
**Distance From Clinic** (n = 598)			.*304 (n = 598)*				.*292 (n = 494)*			.*165 (n = 574)*			.*112 (408)*
<1 Km	90 (27)	245 (73)		161 (60)	69 (26)	40 (15)		290 (91)	29 (9)		163 (73)	53 (24)	
1–5 Km	33 (30)	78 (70)		53 (55)	33 (34)	11 (11)		94 (85)	16 (15)		60 (72)	22 (27)	
6 Km or more	33 (22)	119 (78)		81 (64)	27 (21)	19 (15)		125 (86)	20 (14)		79 (77)	17 (16)	
**Weekly Family Income** (n = 455)			.*619 (n = 455)*				.*619 (n = 455)*			.*564 (n = 436)*			.*990 (n = 295)*
<600 Bs	72 (24)	225 (76)		134 (57)	62 (27)	38 (16)		250 (88)	33 (12)		152 (76)	48 (24)	
600–1200 Bs	32 (28)	83 (72)		58 (64)	22 (24)	11 (12)		95 (86)	16 (14)		49 (75)	16 (25)	
> 1200Bs	9 (21)	34 (79)		18 (55)	36 (13)	3 (9)		35 (83)	7 (17)		23 (77)	7 (23)	
**Education** (n = 633)			.*092 (n = 633)*				**.*05 (514)***			.*346 (n = 607)*			.*930 (n = 422)*
<9 years	75 (28)	194 (72)		117 (52)	70 (31)	38 (17)		228 (91)	23 (9)		143 (73)	44 (23)	
9–11 years	54 (31)	123 (70)		91 (64)	33 (23)	19 (13)		154 (88)	22 (13)		79 (74)	23 (21)	
12 or more	39 (21)	148 (79)		98 (67)	33 (23)	15 (10)		156 (87)	24 (13)		90 (75)	27 (23)	
**Marital Status** (n = 636)			.*389 (n = 636)*				.*401 (515)*			**.*001 (n = 121)***			.*235 (n = 429)*
Single	33 (26)	93 (74)		63 (64)	27 (27)	9 (9)		114 (93)	9 (7)		57 (69)	26 (31)	
Married/consentual union	91 (24)	289 (76)		179 (59)	84 (28)	42 (14)		308 (84)	58 (16)		193 (77)	58 (23)	
Divorced/seperated/widowed	39 (30)	91 (70)		312 (61)	134 (26)	69 (13)		115 (95)	6 (5)		67 (71)	28 (29)	
**Occupation** (n = 650)			***0*.*05 (n = 650)***				.*721 (528)*			.*121 (n = 624)*			.*160 (n = 437)*
Blue collar	45 (22)	158 (78)		96 (60)	39 (24)	24 (15)		168 (87)	25 (13)		89 (73)	33 (27)	
White collar	12 (25)	36 (75)		23 (64)	9 (25)	4 (11)		38 (81)	9 (19)		27 (82)	6 (18)	
Professional	9 (13)	58 (87)		31 (62)	13 (26)	6 (12)		57 (88)	8 (12)		34 (81)	8 (19)	
Other	15 (28)	39 (72)		28 (62)	15 (33)	2 (4)		44 (83)	9 (17)		30 (86)	5 (14)	
Not working/retired	89 (32)	189 (68)		137 (58)	64 (27)	37 (15)		244 (92)	22 (8)		143 (70)	62 (30)	

^a^ percentage of sample per response

**Table 3:** Number of NCDs & occupation by age.

	n (%)^a^	n (%)^a^	n (%)^a^
	Age <45	Age 45–65	Age >65
**# of NCDs**			
1	150 (73)	134 (44)	48 (34)
2	34 (16.5)	78 (26)	38 (27)
3	22 (11)	91 (30)	39 (56)
**Occupation**			
Blue Collar	63 (31)	113 (37)	27 (19)
White Collar	20 (10)	25 (8)	3 (2)
Professional	28 (14)	34 (11)	5 (3)
Other	22 (11	24 (8)	8 (6)
Not Working/Retired	73 (35)	106 (35)	99 (70)

^a^ percentage of sample per response

**Table 4:** Adjusted odds ratios from logistic regression on 4 outcomes of interest.

*Outcome*	*PHQ (Modeled = Depressed)*	*PACIC (Modeled = Med/High)*	*AUDIT (Modeled = Mod/high)*	*Morisky (Modeled = Mod/high)*
*Variable*		* *		
	AORs (95% CI AORs)	AORs (95% CI AORs)	AORs (95% CI AORs)	AORs (95% CI AORs)
**Age (years)**				
45 to <65 vs <45	—	**2.14 (1.35, 3.40)**	**0.42 (0.24, 0.73)**	1.28 (0.71, 2.31)
		Youngest → lower perceptions	Younger → higher use	
≥65 vs <45	—	1.72 (0.99, 2.97)	**0.22 (0.11, 0.53)** Older → lower use	**2.58 (1.39, 4.78)**
				Older → higher adherence
**Gender**			** **	
Male vs Female	**0.59 (0.39, 0.90)**	—	**4.71 (2.73, 8.06)**	—
	Males → less depressed		Males→ higher use	
**Health Literacy**		—		
Low vs Normal	—	—	—	**0.63 (0.41, 0.98)**
				Low Literacy → lower adherence
**Occupation**				
Blue collar vs Not working/retired	**0.58 (0.37, 0.90)**	—	—	—
	Not Working/Retired → more depressed			
White collar vs Not working/retired	0.84 (0.41, 1.73)	—	—	—
Professional vs Not working/retired	**0.38 (0.18, 0.81)**	—	—	—
	Not Working/Retired → more depressed			
Other vs Not working/retired	0.56 (0.26, 1.18)	—	—	—
**Education**		** **		
<9 vs ≥12	—	**1.63 (1.03, 2.58)**	—	—
		Higher Education → Lower Perceptions		
9–11 vs ≥12	—	1.07 (0.65, 1.76)	—	—

**Bolded ORs** are significant predictor variables (95% CI does not include 1).

#### Sociodemographic characteristics by health behaviors (chi-square tests; See Table 2)

The Chi-square analysis conducted for question 2 illustrates that depression varied significantly across gender groups (p < .01). Thirty percent (30%) of all females in the sample were shown to be depressed, as compared with 20% of the males per the PHQ. Gender was also shown to have a relationship with moderate to heavy alcohol use (p < .0001; measured by the AUDIT), where 21% of males were users of alcohol at a moderate or high level, versus 6% of females who reported moderate to high use. The highest rate of alcohol consumption occurs in those under the age of 45 (17%) while the lowest rate is in those <65 years of age (6%). Subjects who were married or in a consensual union had significantly higher rate of moderate to high levels of alcohol use (16%) as compared to single respondents (7%) and those who were divorced, widowed, or separated (5%), however this marriage relationship fails to retain significance in the adjusted logistic models presented later.

The proportion of adherence to medication regimens decreased as age decreased (p < .001). Based on the Morisky scale, 82% percent of those younger than 45 years of age reported the lowest level of adherence, with 77% of those aged 45–65 and 62% of those 65 and older falling into this category. As noted earlier, across all three age groups, forgetting to take their meds was the primary contributor to low medication adherence, with 81% those under 45 reporting forgetting to take medication regularly. Younger people (< 45 years of age) also reported feeling “inconvenienced” by having to take medications (63%). Adherence to medication regimes decreased as health literacy decreased, with 78% of those with low health literacy (n = 319) also reporting low adherence to medications (p < .05).

Education was significantly related to the PACIC measure of perceived care quality. As level of education increased, the percentage of perceived quality of care decreased. Of those with 12 or more years of education, 67% ranked their perception of care quality as low as compared to 52% of those with less than 9 years of schooling. Age was also significantly related to perceptions of care quality. While greater than 50% in all three age groups rated their perceptions of care quality as low, those under the age of 45 reported the lowest perception of quality care (73%) overall. Fifty-four percent (54%) of those between the ages of 45–65, and 57% of those over the age of 65 also rated their perceptions of care quality as low. Finally, occupation was significantly related with depression (p < .05; n = 650). Within the 5 occupation groupings, those who were retired/not working reported the highest level of depression (32%) and professionals reported the lowest level of depression at 13%.

#### Number of NCDs & occupation by age (See Table 3)

A further breakdown of number of NCDs and occupation by age was conducted using a bivariate analysis. This analysis showed that the number of chronic diseases by age varies over the 3 age groups, with those younger than 45 years having the highest percentage of a single chronic disease (73% versus 44% in the middle age group, and 34% in those over 65). As might be expected, those with the highest percentage of 3 or more NCDs were those over the age of 65 (56%). In regards to employment, as expected, those over the age of 65 had a higher rate of “not-working/retired” (70%). However, both of the younger age groups (<45 and those 45–65) also reported high rates of unemployment (35%). When education is broken down by age, those over 65 had the least amount of education, with 68% reporting less than a 9^th^ grade education.

### Logistic regression modeling

As reflected in Table 4, ten of the relationships emerged as significant (per estimated adjusted odds ratios), nearly mirroring the Chi-square analyses (Table 2) and demonstrating their independent effects.

Depression: The regression model of the depression outcome confirmed that men were less likely to be depressed than women (95% CI AORs: 0.39, 0.90) taking all other predictor variables into account. As expected, blue collar & professional groups were 42% & 62% less likely to be depressed compared to the not working/retired group in this study cohort.

#### PACIC

The odds of higher perception of care quality was 2.14 times higher for 45–65 age group than under the age of 45 (95% CI AORs: 1.35, 3.40). Education also emerged as a significant predictor for the PACIC; those respondents who had less than 9 years of education were 1.63 times more likely to have higher perception of care quality than who had ≥ 12 years of education.

#### AUDIT

Age and gender were independently related to moderate to high levels of alcohol use as measured by the AUDIT scale in the regression models. Those who were in the youngest age group (<45) had the highest level of alcohol consumption, being 4.55 times more likely to be moderate/high alcohol users than those in the ≥ 65 age group. Men were 4.71 times more likely to drink than women.

#### Medication adherence

The results of the regression analysis demonstrated that both age grouping and health literacy were independently related to medication adherence as measured by the Morisky. Those respondents who were ≥65 were 2.68 times more likely to have higher medication adherence than the youngest age group (<45). In addition, those with low health literacy were 37% more likely to have a lower level of medication adherence than those in the normal health literacy group.

### Study question 3: Health behaviors, PACIC, & chronic disease

Research question 3 is addressed in Table 5 which reports the analytical results for logistic regression models fitted for four health related dependent measures (PHQ, Morisky, AUDIT, & PACIC) and the five most prevalent NCDs (depression, diabetes, hypertension, high cholesterol, & arthritis). Depression was not used as a predictor variable in the model for PHQ.

**Table 5:** Regressions by number and type of NCDs.

Variable	PHQ (Modeled = Depressed)	PACIC (Modeled = Med/high)	AUDIT (Modeled = Mod/high)	Morisky (Modeled = Mod/high)
	AOR (95% CI AOR)	AOR (95% CI AOR)	AOR (95% CI AOR)	AOR (95% CI AOR)
Arthritis (0 vs 1)	**0.61 (0.38, 0.97)** Arthritics → higher depression	0.78 (0.49, 1.25)	1.07 (0.52, 2.19)	0.69 (0.40, 1.18)
Diabetes (0 vs 1)	0.80 (0.54, 1.20)	**0.41 (0.27, 0.60)** Diabetics → higher perception care quality	1.39 (0.76, 2.53)	0.85 (0.53, 1.34)
High Cholesterol (0 vs 1)	1.37 (0.86, 2.18)	1.00 (0.65, 1.54)	1.05 (0.55, 2.01)	0.75 (0.45, 1.24)
Hypertension (0 vs 1)	0.91 (0.62, 1.35)	0.93 (0.64, 1.38)	1.42 (0.80, 2.54)	0.81 (0.51, 1.28)
Depression (0 vs 1)	N/A	**0.60 (0.38, 0.93)** Higher perception → lower depression	0.94 (0.52, 1.69)	**0.48 (0.27, 0.84)** More depressed, less adherent
Number of NCD†(continuous)	**2.48 (2.05, 3.01)** Higher # NCDs → higher depression	**1.19 (1.02, 1.39)** Higher # NCDs → higher perception of care quality	0.82 (0.65, 1.05)	1.09 (0.91, 1.32)

**Bolded ORs** are significant predictor variables (95% CI does not include 1).

† Separate logistic model was fitted for number of NCD.

A separate regression was used for analysis of the outcome variables PHQ, Morisky, AUDIT, and PACIC and the predictor variable (continuous) of number of chronic diseases (NCD). See Table 5.

#### Regression by type of NCD

The odds of being depressed was 39% lower for individual without a diagnosis of arthritis (0.38,0.97). Respondents with diabetes had higher odds of rating the quality of their care on the PACIC as higher (0.27,0.60) when compared with non-diabetics. Quality of care perceptions were higher in those who were not depressed (0.38, 0.93) while those with depression also had higher odds of having lower levels of medication adherence as measured by the Morisky. Finally, none of the types of chronic diseases were significant factors for predicting the AUDIT outcome.

#### Regression by number of NCDs†

The regression analysis that was used to examine the results of the number of NCDs (continuous) on the outcome variables of the Morisky, AUDIT, and PACIC revealed that the odds of being depressed was 2.48 times higher for a one-unit increase in the number of chronic disease. As shown in Table 5, for each additional unit (number) of a chronic disease the odds of a moderate to high PACIC outcome increased by 1.9.

## Discussion

Using survey data from several primary care settings in La Paz, Bolivia, we conducted an exploratory analysis of four categories of variables including personal characteristics, disease status, the health related behaviors of alcohol use and medication adherence, and the patient’s perception of healthcare quality. Correlation and multiple regression analyses were conducted to examine these relationships. The surveys themselves provide a portrayal of the sociodemographic characteristics and perceptions from individuals with one or more non-communicable diseases (NCDs) in an urban setting within a low-income nation. These results shed light on the public health challenges created by the escalating incidence of NCDs [33] and can be used to inform future interventions[33]

Many respondents in this study had low levels of education, income, employment, and health literacy. These statistics were of no surprise, as we expected a higher number of participants to have lower levels of education, to be employed in mostly blue collar jobs, and to have low levels of health literacy in keeping with prior findings in the region. [6, 10–12] In relation to income, we found that the majority of respondents (65%) had weekly combined family incomes of less than $87.00 USD. In understanding income and poverty in Bolivia, one must note that approximately 83% of all Bolivian wage earners fall outside of the formal income sector.[34] This makes the determination of income tenuous. It is plausible, therefore, that the 30% in our sample who declined or were unable to answer the weekly family income question could be related to non-alignment of the query with the societal norms of employment (e.g. prevalence of the informal economy, self-employment, and seasonal activity) in the region. Alternatively, considering the lack of safety net programs such as disability and unemployment insurance in Bolivia, and the very low percentages of the population that receive pensions in general, the 30% for which no income was recorded may be a close reflection of reality [10]. An excellent overview by Howe et al., suggests several alternative methods for measuring income in LMIC such as participatory wealth ranking (PWR), or the use of other subjective measures of wealth such as the Economic Ladder Question, measuring the perceptions of the adequacy of income to meet household needs, and perceived consumption adequacy [10]. These suggested methods open the door for the consideration of alternative income measures for future studies. Because the links between illness and poverty, particularly in low and middle income countries (LMICs), are well known.[10, 35] increasing allocation of resources towards improving health services is an obvious solution. However, the complexity and seemingly intractable nature of social determinates make the path forward extremely difficult.

The results of the PHQ-8 screening showed that 26% of the population had a depressive disorder, particularly in women and older participants. Moreover, the regression analysis (Table 3) shows that for every additional NCD, the odds of being depressed increased by a factor of nearly 2.5. This would seem logical since women and elderly have higher levels of depression, however, the relationship to the number of NCDs is an important one. Mental illness, particularly depression, is characteristically undiagnosed and underreported in Bolivia, somewhat related to cultural norms/stigma and an extreme shortage of mental health providers [18]. Bolivia ranks the lowest of six South American countries in regards to mental health services, with approximately 1.6 psychiatrists for 100,000 patients and only 0.2% of the total health care budget allocated specifically for mental health care [18]. Combining these facts with increasing NCDs, aging citizenry, high national rates of domestic violence (47%) increasing youth suicide, and alcohol consumption as one of the leading causes of hospitalization [9], the undercurrent of unmet mental health needs seems quite strong. Perhaps we should not have been surprised at the low number of reported diagnoses of depression as compared to the higher numbers detected in our study when we conducted the screening PHQ. Regardless, the state of mental health services and the downstream effects on the health of Bolivian society is an area of needed attention.

The markedly low levels of medication adherence across all age groups, but most notably in those under the age of 45, illustrates both an important challenge and an opportunity for intervention. Our study also showed that depression is significantly related with lower medication adherence as well, emphasizing our prior point about the importance of mental health services for Bolivians. Medication adherence is critical to achieving optimal health outcomes, prompting the WHO to assert that improving adherence has a “greater effect on health than improvements in specific medical treatments” [36]. It is important to note, however, that medication adherence is global problem, with an estimated 50% of patients in developed nations failing to take medications as prescribed [37]. The problem is compounded in low-income countries like Bolivia however, where low drug supply and counterfeit medications interact with poverty, culture, and geography to potentiate the problem. Importantly, current studies [36, 37] show us the important interactions between patient characteristics (literacy, attitudes, involvement in care decisions, age) provider behaviors (communication barriers, patronistic attitudes) and health systems realities (access, cost, availability) that must be taken into account when trying to improve medication adherence. These findings lend support to the PAHO advisory for improving care by increasing patient-centricity, engaging patients in the decision-making process, and improving care quality by adopting the general tenets of the Continuity of Care Model (CCM).

Across the entire sample, 60% of the subjects reported their quality of care as “low” as measured by the total score of the PACIC. Significant relationships emerged between PACIC scores and those under the age of 45, those with higher education, and those with increasing numbers of NCDs. Those with higher levels of education had lower perceptions of the quality of their care as did those in the younger age group (<45). While the reasons for this are unclear it may illustrate that those with a higher education are aware that their primary care is not equitable with other standards around the world [38, 39]. Additionally, younger respondents may also be less accepting of the status quo and more likely to express negative perceptions than those from prior generations, elevating their belief that the quality of the care they receive is sub-par. Finally, although not measured in our study, the ubiquity of cellular connectivity and use of social networking technologies amongst the younger sets may be raising awareness of health inequalities.

Viewing the glass as “half-full” and considering that our sample showed that the younger group had higher levels of education than either of the other two are groups (and yet had the worst perceptions of care quality), could illuminate a segment of the population that might be more willing to engage in innovative interventions targeting both improving self-management of their NCD and/or increase their proactivity in engaging with care providers. This is particularly important when one considers the potential of mHealth approaches to NCD management and the importance of patient engagement as a method to improve engagement in and perceptions of quality care. These findings are supported by the research by Schmittdiel et al. [40] who found that higher scores on the PACIC (perceptions of care quality) are often correlated with increased exercise, higher perceptions of the value of laboratory assessments, and increased receptivity to counseling on how to better care for themselves at home. We believe that this has implications for the structure of future interventions that are planned in this population such as targeted and tailored motivational messaging via cellular telephony, coaching on techniques for engagement in patient-provider encounters, or short messaging service (SMS) reminders regarding healthy behaviors. While perceptions of care quality were low across all groups, opportunities to change may exist, particularly in younger NCD patients in Bolivia. It is important to note however, that adopting the CCM can be accomplished using many different approaches, of which mHealth is single example, and not a panacea. The foundation of “patient-centered care” requires adapting and accommodating the needs, cultures, and beliefs of the individual.

Our results showed that those patients who were diabetic had significantly higher perceptions of quality of care as measured by the PACIC when compared to non-diabetic NCD patients as did those who had higher numbers of comorbidities. In regards to diabetes specifically, perhaps this is related to the pressing and notable impacts of diabetes disease management in relation to feelings of wellness and the dependence on medical intervention that diabetes often brings. Unlike hypertension, which is often called the “silent killer,” or the frequently denied depressive symptoms, those with diabetes often report feeling physically unwell when they are unable to manage their disease. In addition, diabetics and those with numerous comorbidities require consistent management and monitoring over time to avoid a crisis and/or hospitalization, which may contribute to a closer working relationship with care providers. Perhaps the increased frequency of visits positively influences perceptions of care quality; however, this remains to be explored.

Our data also showed that that younger people drink more, particularly those who are male. The impact of age and gender on alcohol habits cannot be denied. Our models failed to find any relationship between the number or types of chronic diseases and the odds of increased drinking habits, and the original significance found between marital status and increased drinking dissolved when gender and age entered into the equation. Alcohol use amongst younger males, even those with NCDs is not surprising and is reflective of much larger global trends. The most revealing aspect of alcohol use in our sample however is related to the low percentage who reported any use of alcohol at all. Our data does not align with larger population studies that show moderately high levels of alcohol use in Bolivia. As referred to elsewhere in this paper, self-report data can be far from reality, especially when answering questions that are sensitive or that go counter to what the participant believes the surveyor wants to hear. The low rates of alcohol use reported here are suspect, although the naïve might interpret this as a sign of better health management amongst NCD patients. We have our doubts.

The low rate of medication adherence across the sample is also common to that found around the globe[36, 37] and several relationships have been discussed earlier. Focusing on the more detailed data regarding reasons for low adherence “forgetfulness” emerged as the post prominent, both while the medication was at hand and when travelling away from home. “Forgetting” may bring to mind issuing reminders–however reminders to take medications will only be successful however when the medication is accessible, and the literature shows that medication accessibility has many facets.[41] Forgetting to carry medications along when travelling outside of the home is but one of those dimensions. Issues such as affordability, availability, and authenticity of medications in LMICs are additional and well known challenges [41]and ones that are beyond the scope of this article to discuss. Therefore, we can only echo the literature in this regard and refer readers to an excellent review by Christiani et al. [42]. Our study did show however that the poorest medication adherence is in those under the age of 45. This is of particular concern; as they are today’s primary workforce and tomorrows generation of NCD sufferers.

The relationships that emerged in our study between alcohol use, depression, gender, age and occupation illustrate themes that are common across the globe [43]. The findings that Bolivian males have greater rates of heavier alcohol use than do females, that younger people have higher rates of moderate to heavy drinking, and that depression is more highly associated with females echoes the findings of many studies [43–45]. Gender inequality and the myriad of sequela associated with repression of women in Bolivia [34] could contribute to the higher rates of depression in women in this sample, although this gender gap is commonly seen worldwide. It is a complex and toxic mixture.

Finally, our finding that those who are retired or not employed had the highest level of depression, is supported by the work by Paul & Moser [46]. It also aligns with the work of Stiglitz et al. (2014) [47] who documented this effect specifically in Bolivian “subsistence societies” where the reduced ability to produce and transfer resources that have value for people and their families were related to increasing risks for depression. As expected, the highest rate of not working/retired in this sample was found in those over the age of 65. While not surprising in many ways (one would expect lower rates of active employment as people age) the lack of pension plans, low Social Security participation rates, and limited access to comprehensive health and social services further raises the probability of elder depression, particularly in this subsistence society. These factors increase the impetus for monitoring and evaluation of the SPAMM (the Bolivian Program for Health Insurance for the Elderly) discussed earlier and lend additional evidence for substantial increases in funding and services to support mental health care. The high level of unemployment across the board and the downstream effects on employment associated safety nets (pensions, unemployment, disability, Social Security) is worrisome. While readers are reminded of the difficulties presented earlier in regards to determining true income and employment status in LMIC, the high unemployment rate does not bode well for the future as generations ag, NCDs increase and the safety nets fray.

The interactions between gender, depression, alcohol abuse, and employment status, require further analysis. It will be necessary to better understand the interplay among these variables—as the diverse cultural fabric of La Paz shapes them—to design interventions that will improve the health status of Bolivians with chronic disease. The call by PAHO to adopt the principles of the Chronic Care Model and to deeply engage patients in the care process will be vital ingredients to disrupt the startling projections for NCDs in Bolivia.

### Limitations

This study sheds light on the demography, health status, and health behaviors of chronic disease sufferers in La Paz, Bolivia, but some limitations of the study should be noted. Our research is based on self-reports from a broad cross sectional sample of patients seeking care for chronic disease across 6 different health care settings in the region of La Paz, Bolivia. Our study was conducted in a setting where the population is heavily indigenous and instability in the political and healthcare sector exists. These factors make the findings somewhat specific to the region, although it should be noted that many of our findings were supported by other studies conducted in similar low resource settings. Conducting survey-based research in these settings is inherently difficult. Participants are wary and suspicious of answering numerous questions. We believe this, and the impact of trying to collect 2 surveys on the same participant during a healthcare visit, may have contributed to a lower rate of complete surveys for the final analysis (n = 651).

Self-reported health data are prone to misrepresentation. This could be particularly true for drinking behaviors, reported income, and the true extent and nature of NCDs reported. Because the literature shows high rates of depression and alcohol use in Latin America, our data may be underestimating their true prevalence. This in turn may have obscured more powerful relationships between depression and perceived quality of care, alcohol use and medication adherence. The impact of mediating factors, confounders, and non-diagnosed mental illness and their impacts on other health behaviors were not examined in this study. It is unquestionable that one of the leading causes of chronic disease burden around the globe is mental/behavioral disorders[48]. As was noted earlier, we acknowledge that the variable of weekly reported income in this study could be problematic due to the nature of the question that was used to measure wealth and the difficulty in assessing this dimension in LMICs. Finally, while we used well-established and Spanish-language tested tools, the plurinational composition of the Bolivian people makes it difficult to totally fine-tune the instruments to the population.

## Conclusion

Our study has illustrated many of the vulnerabilities of the NCD patient population in La Paz and has illuminated some of the challenges that face the primary care system in the region. The complex intertwining of the social determinants of health, social risk factors, health system structure, and behavioral/psycho-social issues presents major obstacles to the management and prevention of NCDs in low and middle income countries. This is not unique to Bolivia.

Our results point to factors that must be accommodated in any NCD-related interventions. Communications with these individuals must be comprehensible to them; because these diseases are chronic, interventions that recur must reflect costs these individuals can bear. Gender disparities and the impact of an aging population add pressures that must be addressed. Difficult choices between food, shelter, and medications will most likely result in decreasing rates of medication and preventive care adherence, particularly in Bolivia where safety nets such as unemployment insurance and social security are weak. Gender based issues are omnipresent. To stem the tide of NCDs in Bolivia will require concomitant efforts to gain a much deeper understanding of mental health concerns, a marked increase in mental health services, and the provision of contextually and culturally sensitive mental health care. A change in the culture of patient-provider interactions that include a greater level of engagement and shared decision-making in the context of care is an aspect that must be adopted across the spectrum–from policy makers to educators to administrators and clinicians.

Opportunities exist for change. Tapping into increasingly savvy and connected younger groups who reported feeling “inconvenienced” by their disease state could be a valuable starting point. Combining methods to increase the public awareness of the consequences of low medication adherence with efforts to improve health behaviors by using public media and cellular telephony has been shown to have marked impact in other NCD-ridden LMICs. [33] Adopting and enforcing strong data collection, management, and evaluation is reliant upon robust information technology. A milestone that cannot be measured can be neither met nor missed. Changing the culture of caring in the healthcare industry, particularly to increase the acceptance and use of patient-centered care, is something that may benefit from Bolivia’s emergence after decades of strife. Bolivia’s overall economic improvement and increasing political stability is noted, as is a general sense of optimism and national pride [49]. The glass may be half full.

The combinations of all of these challenges are daunting to be certain; however, enhanced public and community health efforts coupled with concerted efforts to improve primary care are necessary foundations need to change the status quo. Uncontrolled NCD growth is threatening to crush emerging economies, requiring that effective and cost-efficient interventions be well-planned and well-executed. Siloed efforts to correct one dimension (i.e. medication availability) without the broader focus needed in other problem areas (i.e. levels of poverty that preclude medication purchases) will result in a plight similar to that of Sisyphus and his futile labor [13].

These results from this cross-sectional study support the PAHO recommendations for strengthening the primary care systems in Bolivia and lay the groundwork for examining the financing, the structures, and the processes of care provided to patients with NCDs in the region. Patient-centered care, where shared decision-making and patient engagement is the norm, is foundational to address the challenges of NCDs in areas like LaPaz.
